# Dystonia: Insights into Mechanisms and Novel Therapeutics

**DOI:** 10.1007/s11910-025-01479-7

**Published:** 2026-01-10

**Authors:** Ivana Dzinovic, Michael Zech

**Affiliations:** 1 Institute of Human Genetics, School of Medicine and Health, Technical University of Munich, Munich, Germany; 2 Institute of Neurogenomics, Helmholtz Zentrum München, Munich, Germany; 3 Institute for Advanced Study, Technical University of Munich, Garching, Germany; 4 Institute of Neurogenomics, Helmholtz Zentrum München, Deutsches Forschungszentrum für Gesundheit und Umwelt (GmbH), Ingolstädter Landstraße 1, 85764 Neuherberg, Germany

**Keywords:** Dystonia, Genetics, Pathogenesis, Molecular pathways, Therapeutics

## Abstract

**Purpose of Review:**

Dystonia is a highly heterogeneous movement disorder with complex molecular underpinnings. This review aims to synthesize insights into pathophysiological mechanisms driving dystonia with emphasis on latest advances.

**Recent Findings:**

In recent years, key molecular pathways in dystonia have been elucidated, among them: aberrant transcriptional regulation, altered protein turnover, nuclear envelope dysfunction, and mitochondrial impairment. Emerging data reveal the interplay and convergence of some of these disease-related processes, highlighting overarching molecular vulnerabilities critical to pathogenesis.

**Summary:**

Deciphering molecular mechanisms underlying dystonia facilitates the stratification of affected individuals into biologically defined subgroups, which will be essential for the development of targeted therapies. Patient assessment based on individual molecular profiles represents a promising avenue for future therapeutic and preventive strategies in dystonia.

## Introduction

Dystonia is a movement disorder characterized by a broad spectrum of clinical manifestations and diverse underlying etiologies. Historical reports suggest its presence since antiquity [1], although formal clinical characterization emerged in the 20th century [2]. The latest consensus defines dystonia as abnormal movements or postures—often twisting, but sometimes tremulous or jerky—that may occur independently or in combination [3].

The clinical presentation is highly heterogeneous, with variation in age at onset, anatomical distribution, temporal pattern, and phenomenology [3]. This variability complicates diagnosis, particularly outside specialized movement disorder centers. Dystonia may present in isolation or as part of a broader neurological or systemic syndrome [3], adding further diagnostic complexity.

Although brain MRI may reveal abnormalities in a subset of patients, most exhibit no overt structural changes [2]. Instead, current models conceptualize dystonia as a disorder of motor network connectivity [4]. Investigations of monogenic forms of dystonia have begun to identify the molecular pathways that contribute to these aberrant circuits.

Advances in high-throughput sequencing technologies revolutionized human genetics, broadening access to testing and enabling the discovery of numerous gene–phenotype associations. These developments profoundly influenced dystonia research, where such approaches are now pivotal in elucidating its heterogenous genetic architecture. A recent large-scale study of coding regions in 1,825 dystonia pedigrees identified 205 distinct genetic causes, the majority of which involved genes critical for brain development [5]. These findings underscore the extensive molecular diversity underlying dystonia, including contributions from ultra-rare variants and genetic syndromes.

Current treatments for dystonia focus on symptom management, including botulinum toxin injections, pharmacological therapies, surgical approaches—such as deep brain stimulation, and supportive physical therapies [2]. However, disease-modifying treatments remain a major unmet need. Novel gene-based and small molecule therapies are emerging [6], but their successful application will depend on identifying molecular targets and tailoring interventions to the individual patient’s underlying specific pathology.

In this review, we focus on recent advances in the understanding of molecular pathways implicated in dystonia, including altered gene expression, impaired protein degradation, nuclear envelope dysfunction, and mitochondrial abnormalities. These pathways were selected based on emerging newer evidence supporting their relevance to the pathophysiology of dystonia. While other mechanisms—such as dopaminergic dysregulation, abnormal calcium signaling, synaptic transmission defects, heavy metal accumulation, endo-lysosomal dysfunction, and altered purine metabolism—are also implicated, a detailed discussion of these exceeds the scope of this manuscript and the readers are referred to comprehensive reviews covering those topics [7–10]. Finally, we examine how recent findings reveal convergence across distinct genetic forms of dystonia, and explore how this knowledge may potentially inform future therapeutic strategies.

## Aberrant Transcriptional Regulation Underlies Dystonia

**Fig. 1:**
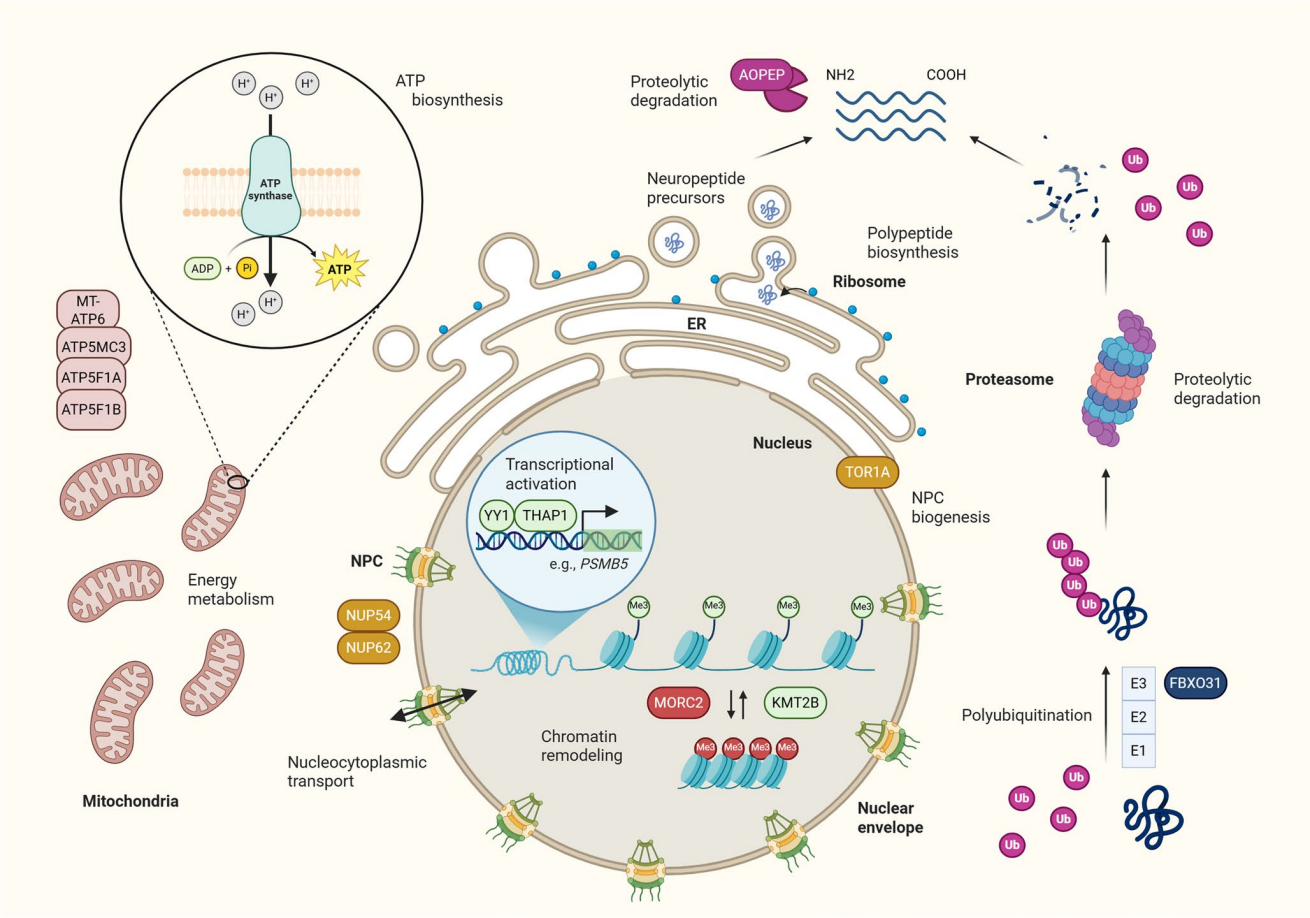
Emerging molecular pathways underlying dystonia. Genes associated with dystonia (indicated by boxes) participate in critical cellular functions ranging from regulation of gene expression within the nucleus (*THAP1*, *YY1*, *KMT2B*, and *MORC2*), maintenance of nuclear envelope integrity (*TOR1A*, *NUP54*, and *NUP62*), mitochondrial energy metabolism and ATP synthesis (*MT-ATP6*, *ATP5MC3*, *ATP5F1A*, and *ATP5F1B*) to protein quality control and proteolytic degradation (*THAP1*, *FBXO31*, and *AOPEP*). Further functional details and the impact of dystonia-linked genetic variants are discussed in the main text. ADP = adenosine diphosphate; ATP = adenosine triphosphate; E1 = ubiquitin-activating enzyme; E2 = ubiquitin-conjugating enzyme; E3 = ubiquitin ligase; ER = endoplasmic reticulum; Me = methyl group; NPC = nuclear pore complex; Pi = inorganic phosphate; Ub = ubiquitin. Figure was designed with BioRender.com

A tightly regulated spatiotemporal pattern of gene expression is essential for proper brain development. One of the initial steps in this multilayered process is the packaging of DNA around histone complexes, which regulates DNA accessibility to the transcriptional machinery. Chromatin remodelers, histone modification enzymes, and components of transcriptional complexes are crucial at this stage, with perturbations in genes encoding them often leading to aberrant neurodevelopment [11]. The resulting phenotypic spectrum frequently includes dystonia, which often co-occurs with additional symptoms [12, 13]. On the other hand, *THAP1*- and *KMT2B*-related disorders represent two prominent examples associated with transcriptional dysregulation that often feature isolated or seemingly isolated dystonia [14] (Fig. 1).

*THAP1* (THAP domain-containing protein 1) codes for a zinc finger transcription factor with important roles in early developmental stages, such as stem cell survival and differentiation [15]. Missense and loss-of-function (LoF) variants that impair DNA binding, cause THAP1 mislocalization, or disrupt its interaction with cofactors, result in isolated dystonic abnormalities with incomplete penetrance [8, 14]. According to studies in cortical neurons derived from human induced pluripotent stem cells (iPSCs), pathogenic alterations induce transcriptional dysregulation, which is more pronounced in manifesting than in non-manifesting mutation carriers [16]. Neurodevelopmental genes and genes involved in myelination seem to be particularly affected [17]. While murine models corroborate myelination defects [17, 18], human *THAP1* mutation carriers typically do not display gross abnormalities on brain MRI, thereby underscoring the need for further research into the downstream effects of *THAP1*-related transcriptional perturbations in the human brain. Only recently, the zinc finger transcription factor encoded by *YY1* (yin and yang 1), involved in oligodendrocyte maturation and initiation of myelin formation, was linked to a neurodevelopmental disease with variable dystonia [19–22]. Interestingly, YY1 and THAP1 interact closely and co-regulate a joint set of genes, with some pathogenic *THAP1* variants preventing this interaction and the formation of an active transcription complex [18].

*KMT2B* (lysine-specific methyltransferase 2B) encodes an epigenetic regulator that introduces methyl groups to the lysine residue (K4) of histone H3 associated with active transcription. Trimethylation of H3K4 (H3K4me3) is particularly enriched at active promoters, while a single methyl group (H3K4me1) is often found around active enhancers [23]. KMT2B´s ubiquitous expression includes brain tissue, where it plays an important role in stem cell differentiation and motor circuit maturation [24]. Interestingly, variants in other genes from the histone-specific methyltransferase family almost exclusively lead to global developmental disturbances [25], while monoallelic *KMT2B* disruption induces predominant movement abnormalities, sometimes co-occurring with variable developmental comorbidities, and rarely resulting in a purely delayed developmental phenotype [14, 26]. The reason behind this discrepancy is still unknown. An important breakthrough has recently been achieved by independent research groups who identified a non-random pattern of DNA hypermethylation in peripheral blood - an episignature - resulting from *KMT2B* pathogenic variants [27–29]. The observed pattern proved to be highly specific for *KMT2B* disturbance and enabled the reclassification of ambiguous genetic variants [27]. Furthermore, some characteristics of the disease, such as the age at onset, could be predicted based on the epigenetic profile [27]. DNA methylation profiling has gained prominence as further illustrated by unique episignatures for *YY1-* [30], and also *MORC2-*associated disorders [31]. Pathogenic variants in the chromatin remodeler *MORC2* (microrchidia CW-type zinc finger protein 2) are mostly missense changes with gain-of-function (GoF) effects that cause a wide clinical spectrum ranging from neurodevelopmental phenotypes to late-onset neuropathy [31]. Intriguingly, the spectrum also includes dystonia [32–34]. Although specific episignatures can be identified, subtle inter-patient DNA methylation differences might contribute to the encountered phenotypic diversity among individuals with a similar genetic defect, suggesting an avenue for further research.

Defective components of the gene-regulation machinery may lead to distinctive changes in transcriptional profiles, as evidenced by differentially expressed genes detected in patient-derived cells with *THAP1* [16] and *MORC2* [31] mutations. Similarly, proper KMT2B functioning is essential for adequate expression of other dystonia-linked genes, such as *THAP1* and *TOR1A*, which were reported to be downregulated in fibroblasts derived from patients with *KMT2B* pathogenic variants [23]. Previously, experimental evidence indicated that *THAP1* regulates its own expression [35] and acts as a *TOR1A* suppressor [36, 37]. Considered together, these findings suggest that altered transcriptional regulation seems to be a unifying pathomechanism for certain forms of dystonia. Experimental work in mouse embryonic stem cells revealed that KMT2B does not just write histone marks, but rather it prevents transcriptional silencing by repelling components of PRC2 (polycomb repressive complex 2) and DNA methyltransferases (DNMTs) [38]. Potential therapeutic approaches focused on methylation modifications could use these novel targets, thereby coming a step closer to treatments aimed at the underlying pathology (Table 1). Epigenetic silencing has been successfully harnessed in the field of oncology, exemplified by the DNMT inhibitors azacitidine [39] and decitabine [40], alongside the PRC2 inhibitor tazemetostat [41]. Nonetheless, given the ubiquitous nature of DNA methylation, precise target selectivity and thorough experimental evidence remain critical prerequisites.

**Table 1:** Potential therapeutic avenues targeting impaired molecular pathways in dystonia

Molecular pathway	Representative Gene(s)	Potential molecular interventions that may deserve further experimental exploration
Transcriptional regulation	*KMT2B*	DNMT inhibition?
		PRC2 inhibition?
Protein quality control	*THAP1*	Proteasomal activation?
	*AOPEP*	M1 aminopeptidase modulation?
Nucleocytoplasmic transport	*TOR1A*, *NUP54*, *NUP62*	Nuclear-cytoplasmic transport modulation?
		Modulation of NPC-associated chaperones?
Mitochondrial function	*MT-ATP6*, *ATP5MC3*, *ATP5F1A*, *ATP5F1B*	Boosting electron transport chain?
		Increasing the levels of ADP and Pi?
		Antioxidants?
		Stimulation of mitochondrial biogenesis?

ADP = adenosine diphosphate; AOPEP = aminopeptidase O; ATP5F1A = ATP synthase F1, subunit alpha; ATP5F1B = ATP synthase F1, subunit beta; ATP5MC3 = ATP synthase membrane subunit c, locus 3; DNMT = DNA methyltransferase; KMT2B = lysine-specific methyltransferase 2B; MT-ATP6 = ATP synthase 6; NPC = nuclear pore complex; NUP54 = nucleoporin 54; NUP62 = nucleoporin 62; Pi = inorganic phosphate; PRC2 = polycomb repressive complex 2; THAP1 = THAP domain-containing protein 1; TOR1A = torsinA

### Defective Protein Turnover and Proteasomal Degradation Emerge as Mechanisms in Dystonia

Ensuring precise regulation of protein quality and abundance is crucial for cellular homeostasis. Central to this process is the proteasome – a multi-subunit complex capable of degrading ubiquitinated proteins in an ATP-dependent manner [42]. While proteasomal dysfunction and consequent accumulation of aberrant proteins have been implicated in numerous human diseases [42], its contribution to dystonia has only recently emerged (Fig. 1).

Despite the evolutionary conservation of the proteasome across all eukaryotes, factors that regulate expression of its subunits under basal conditions have remained elusive for a long time. Two recent back-to-back publications reported that the expression of the core proteolytic subunit β5, encoded by *PSMB5*, falls under direct regulation of THAP1 [43, 44]. Experimentally knocking out *THAP1* caused diminished *PSMB5* expression, decreased proteasomal activity, and subsequent accumulation of ubiquitinated proteins in the cell [43, 44]. Furthermore, by exploring the impact of single amino acid substitutions on *THAP1* activity, studies showed that dystonia-related *THAP1* variants produced a similar effect, providing a mechanistic link between THAP1 dysfunction and impaired proteasomal regulation [44]. Notably, *PSMB5* overexpression was sufficient to rescue the cellular phenotype [43, 44], raising important questions: could genetic variation in proteasome-related genes act as a protective factor, thereby contributing to the well-recognized incomplete penetrance in *THAP1*-related dystonia? Furthermore, might therapeutic modulation of proteasomal gene expression offer a viable strategy for phenotypic rescue?

While these hypotheses still await further investigation, there are other genes involved in the ubiquitin-proteasomal pathway that can be associated with dystonic manifestations, including *UBE3A* (ubiquitin-protein ligase E3) [45], *UBA5* (ubiquitin-like modifier-activating enzyme 5) [46, 47], and *FBXO31* (F-box only protein 31). *FBXO31* has been linked to a rare spastic-dystonic cerebral palsy syndrome [48]; the encoded protein FBXO31 is a constituent of the SKP1–CUL1–F-box protein (SCF) ubiquitin ligase complex that catalyzes the transfer of ubiquitin moieties to substrates destined for proteasomal degradation. The precise role of FBXO31 within the SCF complex has only recently been elucidated. FBXO31 recognizes C-terminal amides, which result from oxidative protein damage [49]. As a consequence, C-terminal amide-bearing proteins are polyubiquitinated and rapidly cleared from the cell [49]. The dominant dystonia-associated missense variant c.1000G > A (p.Asp334Asn) in *FBXO31* [48, 49] eliminates the negative charge in the conserved substrate-binding pocket, thereby impeding the recognition of C-terminal amides and resulting in aberrant targeting of a set of specific protein substrates [49].

Biallelic LoF variants in another regulator of protein turnover, *AOPEP* (aminopeptidase O), have been implicated in isolated dystonia [50]. The protein encoded by *AOPEP* belongs to the M1 aminopeptidase family that processes a variety of polypeptides [51], thereby ensuring proteome homeostasis. Although the precise role of AOPEP awaits further research, data point towards its potential function in selective proteolysis of N-terminal amino acid bonds, a crucial process in the activation of neuropeptides and downstream cellular signaling [52]. AOPEP is particularly enriched in glial cell types [53], such as oligodendrocytes, underscoring its potential role in myelination. This association again highlights myelination impairment as a converging mechanism in the etiology of certain monogenic subtypes of dystonia [18]. On the other hand, computational predictions indicate AOPEP`s involvement in endocytosis and proper functioning of the endo-lysosomal pathway [54], another mechanism recently linked to dystonic manifestations [9].

Taken together, the recent findings emphasize a relevant role of protein quality control pathways in dystonia and highlight directions for future research and, possibly, therapeutic development (Table 1). Peptide-based (e.g., P200, PAP1 (proteasome-activating peptide 1)) and small molecule agonists (e.g., betulinic acid, chlorpromazine) that enhance basal proteasome activity or act as gate-openers are in early development, primarily driven by efforts to treat neurodegenerative diseases [55]. Posttranslational modifications of proteasome subunits have been shown to enhance proteasome function and confer neuroprotection. For example, rolipram, a phosphodiesterase 4 (PDE4) inhibitor, increases brain cAMP levels, leading to activation of protein kinase A (PKA) and phosphorylation of the proteasome subunit Rpt6; this cascade enhances proteasome activity, facilitates clearance of pathological tau, and improves cognitive function in Alzheimer disease models [56]. Directly targeting specific E3 ligases such as FBXO31 offers another regulatory avenue. Additionally, oncology-focused M1 aminopeptidase modulators like bestatin and tosedostat could inspire further exploration of aminopeptidase-targeted therapies [57, 58].

## Dysfunction of the Nuclear Envelope Contributes to Dystonia Pathogenesis

A hallmark of eukaryotic cells is the compartmentalization of genetic material within the nucleus, separated from the cytosol by the nuclear envelope (NE). A growing body of evidence implicates dysfunction of the NE in dystonia pathogenesis.

The NE comprises an outer and an inner membrane, demarcated by the perinuclear space [59]. There is a structural continuity between the outer NE membrane and the phospholipid bilayer of the endoplasmic reticulum (ER), as well as the perinuclear space with the ER lumen, which facilitates the exchange of proteins between the two compartments [59]. Among the proteins that localize to this interface is torsinA, an ATPase of the AAA + superfamily, implicated in protein trafficking, refolding, and degradation [60]. One of the most common forms of early-onset isolated dystonia has been associated with the recurrent deletion of one glutamic acid residue (n. ΔGAG, p. ΔE) in *TOR1A*—the gene encoding torsinA [61]. While wild-type torsinA shuttles between the ER and the nucleus, the ΔE mutant predominantly accumulates in the perinuclear space [62]. Selective bidirectional transport of macromolecules between the nucleus and the cytosol is mediated by the nuclear pore complex (NPC), a large multiprotein structure that spans both membranes [63]. Notably, torsinA deficiency has been linked to perturbed NPC biogenesis, which coincides with the appearance of abnormal inner NE membrane herniations [64, 65]. These bleb-like structures are enriched with nucleoporins (NUPs), the core components of the NPC, as well as molecular chaperones from the HSP40 and HSP70 families, which are essential for the proper folding and remodeling of protein complexes [66]. Furthermore, experimental data from a *Tor1a* ^ΔGAG/+^ mouse model showed that the nuclear proteome is altered under conditions of cellular stress [67], hence linking disturbances in nuclear-cytoplasmic trafficking, proteostasis, and stress response pathways in dystonia etiology.

Remarkably, pathogenic variants in *NUP62* [68] and *NUP54* [69], two major components of the NPC inner transport channel, have been implicated in early-onset movement disorders with dystonia and striatal abnormalities (Fig. 1). Although reported in a limited number of families, these variants are predicted to disrupt protein-protein interactions, leading to downregulation of additional NPC components [69]. These findings provide a rationale for further exploration of impaired intracellular macromolecular trafficking as a mechanism underlying dystonia.

Although speculative, therapeutic strategies targeting nuclear-cytoplasmic transport may deserve investigation (Table 1). Inhibitors of nuclear import [70, 71] and nuclear export [72] may be considered as potential candidates for modulating NE dysfunction. Additionally, targeting NPC-associated chaperones may offer an avenue for intervention; notably, the HSP90 inhibitor ganetespib is currently undergoing clinical evaluation in oncology [73]. Incomplete penetrance of the ΔE *TOR1A* mutation has been consistently observed [2]. Noteworthy, the NE bleb-like structures observed in disease models appear to be transient, suggesting the existence of a neurodevelopmental window of vulnerability [74]. This aligns with clinical observations that individuals who remain asymptomatic beyond the age of 30 are unlikely to develop the disease [2]. The identification of the modifying factors that are active during the vulnerable developmental stage could have an important impact on the mutation carriers, as timely intervention could modify the phenotype towards normal neurodevelopment. Interestingly, the human genome contains paralogs of *TOR1A* which might have compensatory roles. Experimental upregulation of *TOR1B* in model systems seems to ameliorate the phenotype [75], providing another promising avenue for treatment development. The downstream consequences of impaired NPC function have been more extensively explored in other neurologic conditions [76]. Insights gained from these studies may be extrapolated to dystonia, potentially informing the development of novel therapeutic strategies.

## Mitochondrial Impairment and Dystonia

Mitochondria ensure energy supply to sustain proper cellular metabolism. Therefore, it is not surprising that mitochondrial dysfunction often leads to multisystem impairment and complex clinical presentations [77]. Some cell types are particularly vulnerable to mitochondrial perturbations, especially those with high energy requirements, such as neuronal cells. Indeed, mitochondrial impairment has been implicated in a variety of neurologic disorders including dystonia [78, 79].

Recent large-scale exome and genome studies reinforced the significant role of mitochondrial genes in dystonia pathogenesis. These research efforts associated pathogenic variants in both nuclear [80] and mitochondrial DNA [5] with dystonia; affected carriers often displayed additional neurologic symptoms and early disease onset. For example, dystonia is a common feature of Leigh syndrome, an early-onset mitochondriopathy characterized by bilateral symmetrical basal ganglia lesions [81]. It occurs in up to 60% of affected individuals, often spreading to multiple body regions or becoming generalized [82]. Many genes have been implicated in Leigh syndrome, including *MT-ATP6*, which encodes a subunit of mitochondrial complex V [81, 82].

Mounting evidence highlights that especially dysfunction of complex V is causally related to dystonia (Fig. 1). The mitochondrial oxidative phosphorylation (OXPHOS) system consists of five multi-subunit complexes, with complex V representing an ATP synthase [77]. Nineteen different genes code for proteins that constitute this multimer structure [83], with some of them previously implicated in severe clinical syndromes associated with autosomal recessive inheritance [84–86]. While these severe encephalopathies might be accompanied by dystonia [87, 88], reports of mitochondrial deficiency manifesting as isolated movement disorders have been limited until recently. Associations between pathogenic heterozygous variants in *ATP5MC3* [89, 90], *ATP5F1A* [90, 91], and *ATP5F1B* [91, 92] with dystonic phenotypes have renewed interest in the mitochondrial origin of movement disorders [93]. Functional in vitro studies indicated that reduced complex V activity appears to be a common denominator in this group of patients [89–92], likely due to decreased protein levels or impaired subunit interaction leading to multimer misassembly and defective mitochondrial function. Hence, proposed pathomechanisms include both LoF and dominant-negative effects. In contrast, biallelic variants that induce complete absence of the protein tend to result in devastating clinical presentations exceeding isolated movement abnormalities. Precise characterization of variant effects is critical, especially given ongoing drug repurposing efforts [94] where accurate patient stratification is essential.

Insights derived from other diseases may offer a framework for addressing mitochondrial dysfunction in the context of dystonia (Table 1). Potential therapeutic avenues may involve enhancing electron transport chain activity using compounds such as ubiquinone (CoQ10) or idebenone, both of which have demonstrated beneficial effects in cellular models [95, 96], model organisms [97], as well as clinical trials in Parkinson disease [98] and Friedreich ataxia [99]. Another strategy may focus on increasing the availability of essential substrates for ATP synthesis through supplementation with creatine [100] or L-carnitine [101]. Given that impaired ATP production is often associated with elevated reactive oxygen species generation, the benefit from antioxidant therapy could be experimentally explored [102]. Finally, deficits in energy metabolism may be ameliorated by stimulating mitochondrial biogenesis via compounds like resveratrol [103] or pyrroloquinoline quinone (PQQ) [104], although caution is warranted due to the pro-apoptotic potential of resveratrol [105].

## Conclusions

Dystonia is recognized as a phenotypically and etiologically heterogeneous movement disorder, challenging the conventional “one size fits all” treatment paradigm. However, the much-needed tailored therapeutic interventions demand rigorous patient stratification based on molecular and pathophysiological underpinnings.

No singular mechanistic framework accounts for all dystonia phenotypes; rather, overlapping molecular networks contribute to diagnostic and therapeutic complexity. How exactly disruptions in alternative molecular routes result in the same abnormal movement phenotype still remains an open area of investigation. Moreover, variation in the same molecular cascade can give rise to diverse phenotypic outcomes, posing another intriguing line of inquiry. Addressing these questions would require coordinated efforts from researchers across disciplines, spanning basic neuroscience to clinical practice.

Nonetheless, convergent themes are beginning to emerge, notably defects in neurodevelopment and myelination. Supporting this notion, large-scale sequencing cohorts have identified shared genetic factors linking dystonia and neurodevelopmental disorders [106]. These insights advocate for in-depth investigation of early developmental windows to identify actionable vulnerabilities and further delineate the aberrations of neurodevelopment leading to dystonic abnormalities.

Recent advances in monogenic dystonia research have delineated converging molecular disruptions, such as translational dysregulation, impaired proteasomal degradation, NE abnormalities, and mitochondrial dysfunction. A new key example of pathway intersection is the transcription factor THAP1, which orchestrates gene expression [17] and proteostasis by regulating critical proteasomal subunits [43, 44]. This discovery underscores the integration from transcription to proteome maintenance in the context of dystonia. Similarly, TOR1A, traditionally implicated in cellular stress resilience [67], has been linked to NE defects [66], a finding reinforced by the identification of dysfunctional NPC components in dystonia [69]. Furthermore, continued investigation may reveal pathway components which contribute most to dystonic outcomes, as seen with the predominance of complex V deficiency in dystonia-related mitochondrial dysfunction [89–92].

Despite advances in genetics, phenotypic heterogeneity and incomplete penetrance remain major challenges. These complexities make it difficult to predict disease presentations based on genotypic information. Molecular stratification could begin entering clinical practice by combining large-scale sequencing with deep phenotyping and long-term follow-up to refine estimates of penetrance, expressivity, and individual risk. Such stratification would allow clinicians to provide probabilistic risk assessments rather than deterministic predictions, improving patient counselling and monitoring strategies. Though common genetic variants contribute to these phenomena, scientific attention is now also focusing on interactions between genotype and environment. Rigorous and systematic evaluation of environmental modulators within molecularly stratified patient cohorts is imperative to delineate critical triggers and protective modifiers governing disease onset and progression. Ultimately, this information could inform prevention strategies and mitigate disease manifestation. While most approaches remain in the research phase, ongoing advances in sequencing technologies, patient registries, and biomarker identification make the gradual implementation of molecular stratification in clinical decision-making increasingly feasible, offering a realistic path toward more personalized care in dystonia.

The translational application of molecular discoveries may offer potential to redefine dystonia management. A deeper understanding of the underlying pathophysiology is a prerequisite for the identification of robust biomarkers, which have historically been lacking in movement disorders. Notably, epigenetic signatures associated with pathogenic variants in *KMT2B* [27–29], *YY1* [30], and *MORC2* [31] exemplify progress toward prognostic tools relevant to personalized patient counseling. We underscore the imperative to expand access to comprehensive molecular diagnostics in dystonia, by leveraging global consortia to accelerate discovery. The integration of other omics layers, such as high-throughput profiling of the cellular transcriptome and proteome [5], will be pivotal in providing additional functional context for genetic variants and refining diagnostic precision.

Advancing our understanding of the molecular pathways underlying dystonia pathogenesis is critical for the identification of novel therapeutic targets. Investigating the potential of existing pharmacological agents, including those currently approved or in development, through drug repurposing strategies holds potential promise (Table 1). Nevertheless, rigorous scientific validation is imperative, as robust evidence supporting each candidate’s efficacy and mechanism remains to be established. Successful pre-clinical drug screens may not translate into clinical success due to uncertainties surrounding blood-brain barrier penetration, altered pharmacokinetics, and potential off-target effects that could impact tolerability and efficacy. While several of the therapeutic strategies discussed in this review remain more speculative, some avenues show clearer promise. In particular, targeting mitochondrial dysfunction is supported by emerging evidence, with several mitochondria-modulating compounds already in clinical trials for other neurological disorders [98, 99], though none have yet been tested in dystonia-specific context. Nonetheless, growing mechanistic insight and an expanding pool of candidate compounds provide a strong basis for future translational progress, offering cautious optimism for more effective therapies ahead.

In summary, a multidimensional molecular framework is emerging, poised to transform dystonia from a clinically heterogeneous syndrome into a spectrum of biologically defined disorders, each ultimately amenable to targeted intervention. This paradigm shift offers new opportunities for personalized medicine and improved patient outcomes.

## Key References

- Albanese A, Bhatia KP, Fung VSC, Hallett M, Jankovic J, Klein C, et al. Definition and Classification of Dystonia. Movement disorders : official journal of the Movement Disorder Society. 2025.
  - The latest update of the dystonia definition that incorporates guidelines for its clinical and etiological characterization, proposed by an expert panel of movement disorder specialists.
- Zech M, Dzinovic I, Skorvanek M, Harrer P, Necpal J, Kopajtich R, et al. Combined genomics and proteomics unveils elusive variants and vast aetiologic heterogeneity in dystonia. Brain. 2025.
  - Large-scale multi-omics study of a dystonia-patient cohort that highlights vast genetic heterogeneity and the implication of neurodevelopmental genes in the etiology of dystonia.
- Wang Y, Wang Y, Iriki T, Hashimoto E, Inami M, Hashimoto S, et al. The DYT6 dystonia causative protein THAP1 is responsible for proteasome activity via PSMB5 transcriptional regulation. Nat Commun. 2025;16 [1]:1600.
  - Research describing convergence between aberrant transcriptional activity and dysregulated proteasomal function in dystonia.
- Muhar MF, Farnung J, Cernakova M, Hofmann R, Henneberg LT, Pfleiderer MM, et al. C-terminal amides mark proteins for degradation via SCF-FBXO31. Nature. 2025;638(8050):519 − 27.
  - A study that identified the role of FBXO31 within the proteasomal pathway, also highlighting the role of a dystonia and spasticity-associated dominant mutation in protein-turnover alterations.
- Prophet SM, Rampello AJ, Niescier RF, Gentile JE, Mallik S, Koleske AJ, et al. Atypical nuclear envelope condensates linked to neurological disorders reveal nucleoporin-directed chaperone activities. Nat Cell Biol. 2022;24 [11]:1630-41.
  - In vitro study that connects *TOR1A*-associated nuclear envelope defects with impaired nucleoporin functioning.
- Li J, Levin DS, Kim AJ, Pappas SS, Dauer WT. TorsinA restoration in a mouse model identifies a critical therapeutic window for DYT1 dystonia. J Clin Invest. 2021;131 [6].
  - An investigation of TOR1A function during murine neurodevelopment with implications for the timing of therapeutic intervention in dystonia.

## Data Availability

No datasets were generated or analysed during the current study.
